# The effect of taurine supplementation on the renin–angiotensin–aldosterone system of dogs with congestive heart failure

**DOI:** 10.1038/s41598-023-37978-1

**Published:** 2023-07-03

**Authors:** Sara Brethel, Seth Locker, Renee Girens, Paulo Rivera, Kathryn Meurs, Darcy Adin

**Affiliations:** 1 College of Veterinary Medicine, University of Florida, Gainesville, FL USA; 2 College of Veterinary Medicine, North Carolina State University, Raleigh, NC USA; 3 Present Address: Charlotte Animal Referral and Emergency, Charlotte, NC USA; 4 Present Address: Summit Veterinary Referral Center, Tacoma, WA USA

**Keywords:** Heart failure, Translational research

## Abstract

The role of taurine in the treatment of congestive heart failure (CHF) in dogs without systemic deficiency is unexplored. Taurine might have beneficial cardiac effects aside from deficit replacement. We hypothesized that oral taurine supplementation administered to dogs with naturally-occurring CHF would suppress the renin-angiotensin aldosterone system (RAAS). Oral taurine was administered to 14 dogs with stable CHF. Serum biochemical variables, blood taurine concentrations, and comprehensive analysis of RAAS variables were compared before and 2 weeks after taurine supplementation added to background furosemide and pimobendan therapy for CHF. Whole blood taurine concentrations increased after supplementation (median 408 nMol/mL, range 248–608 before and median 493 nMol/mL, range 396–690 after; *P* = .006). Aldosterone to angiotensin II ratio (AA2) was significantly decreased after taurine supplementation (median 1.00, range 0.03–7.05 before and median 0.65, range 0.01–3.63 after; *P* = .009), but no other RAAS components significantly differed between timepoints. A subset of dogs showed marked decreases in RAAS metabolites after supplementation and these dogs were more likely to have been recently hospitalized for CHF treatment than dogs that did not show marked decreases in classical RAAS metabolites. Overall, taurine only lowered AA2 in this group of dogs, however, response heterogeneity was noted, with some dogs showing RAAS suppression.

## Introduction

Dilated cardiomyopathy and degenerative mitral valve disease are the most common heart diseases of dogs^1,2^. Congestive heart failure (CHF) is a common end point of heart disease from either of these conditions and is a major cause of morbidly and mortality in dogs^3^. With rare exceptions, the underlying disease process progresses despite medical management with diuretics, positive inotropes, and renin-angiotensin aldosterone system (RAAS) inhibitors^3^. Many dogs eventually die or are euthanized because of recurrent CHF, and therefore additional strategies and medications to improve the quality and quantity of life for dogs with CHF are needed.

Taurine is a ubiquitous amino acid that is critical for many physiologic functions^4,5^. Systemic deficiency of taurine or its precursors caused by inadequate dietary provision has been associated with a reversible form of dilated cardiomyopathy in both dogs and cats^6–11^. There is a growing interest in the role of taurine for the treatment of CHF in dogs because of these historical reports of taurine-deficient dilated cardiomyopathy and recent reports of non-taurine-deficient diet-associated dilated cardiomyopathy^10,12–16^. Recent reports of non-taurine deficient diet-associated dilated cardiomyopathy leading to CHF in dogs eating high-pulse dog foods have clouded the role of taurine in recovery, because most dogs were supplemented with taurine in addition to diet change, despite not being systemically taurine deficient^15,17,18^. This situation arose because of the time delay for receipt of blood taurine results from laboratories and because of low cost and perceived good safety profile of taurine. Exogenous taurine might have benefits separate from deficit replacement which have not been explored in the setting of naturally occurring CHF in dogs^4,5,19–23^. Taurine has antioxidant effects, positive inotropic effects, and angiotensin II antagonistic effects in rodents, which could explain the benefit of supplementation seen in people with CHF^4,5,19,23^. These cardioprotective effects of taurine have not been evaluated in dogs with naturally occurring CHF but could be a reason for perceived benefit in non-taurine-deficient diet-associated dilated cardiomyopathy. The clinical impact of a safe and inexpensive nutritional supplement that has benefit for dogs with CHF of any cause could be high. Additionally, demonstration of cardiac benefit of taurine supplementation separate from deficit replacement would improve our understanding of the role that taurine supplementation has played in dogs that have recovered from diet-associated dilated cardiomyopathy.

The purpose of this study was to determine the effect of short-term oral taurine supplementation on the RAAS cascade in dogs with naturally occurring CHF. Congestive heart failure causes RAAS activation because of advanced cardiac disease and the diuretic medications required for the treatment of CHF^24,25^, and it was against this background that we sought to evaluate the effect of taurine on the RAAS. We hypothesized that taurine supplementation would globally suppress formation of RAAS metabolites.

## Results

Fourteen dogs met inclusion criteria. The median age was 132 months (range 84–184) and median weight was 8.50 kg (range 4.06–22.70). Represented breeds were Cavalier King Charles Spaniel (4), Dachshund (3), mixed (3), Chihuahua (2), Maltese (1), and Boston Terrier (1). There were 4 spayed female, 8 castrated male, and 2 intact male dogs. The underlying heart disease in all dogs was myxomatous mitral valve degeneration. Six dogs were ACE gene wildtype and 8 dogs were ACE variant positive (3 heterozygous, 5 homozygous). The median time between visit 1(V1) and visit 2 (V2) was 14 days (range 11–20). The median time since last hospitalization was 8.5 days (range 1–265). The median daily oral furosemide dosage was 3.56 mg/kg (range 1.33–4.54) and the median daily pimobendan dosage was 0.61 mg/kg (range 0.46–0.90). Five dogs received other medications, including diphenoxylate (1), amlodipine (1), levetiracetam (2), fish oil (1), prednisone (1), tylosin (1), gabapentin (1), traumeel (1), CoQ10 (1), and potassium gluconate (1). Median oral taurine dosage administered after V1 was 64 mg/kg/day (range 57–123).

Table 1 shows biochemical, taurine, and RAAS data for V1 (before taurine supplementation) and V2 (after taurine supplementation). Body weight decreased between visits (*P* = 0.02). No dogs had low plasma or whole blood taurine concentrations at V1 and the plasma and whole blood taurine concentrations for all dogs were above the reference range at V2 after taurine supplementation. Whole blood taurine concentration increased between visits (*P* = 0.006) but the increase in plasma taurine concentration between visits did not reach statistical significance (*P* = 0.19). Serum sodium concentration increased between visits (*P* = 0.03) but the remainder of biochemical variables did not change.

**Table 1:** Comparison of clinical, biochemical, and renin-angiotensin aldosterone system variables before (V1) and after (V2) oral taurine supplementation. The data are reported as median (range). *P* values are from Wilcoxon matched-pairs signed rank test and bold indicates a statistically significant difference between visits. V1; visit 1, V2; visit 2, ACE; angiotensin-converting enzyme, NEP; neutral endopeptidase, AA2; aldosterone to angiotensin II ratio, PRA-S; plasma renin activity surrogate, ACE-S; angiotensin-converting enzyme surrogate, ALT-S; alternative RAAS pathway surrogate.

Variable	Reference range if known	V1	V2	*P*
Body weight (kg)		8.50 (4.06–22.70)	8.25 (4.06–20.60)	**0.02**
Heart rate (bpm)	60–160	135 (102–180)	140 (120–240)	0.85
Systolic blood pressure (mmHg)	80–160	146 (100–175)	145 (130–180)	1.0
Plasma taurine nMol/mL	60–120	186 (74–368)	219 (153–445)	0.19
Whole blood taurine nMol/mL	200–350	408 (248–608)	493 (396–690)	**0.006**
Bicarbonate (18–28 mEq/L)	18–28	25 (22–29)	24 (21–28)	0.29
Sodium (141.9–150.6 mmol/L)	141.9–150.6	145.2 (139.2–153.0)	148.2 (139.2–149.9)	**0.03**
Potassium (3.8–5.0 mmol/L)	3.8–5.0	4.0 (3.2–4.9)	4.4 (3.4–4.9)	0.07
Chloride (107.8–117.1 mmol/L)	107.8–117.1	101.5 (92.8–129.0)	107.8 (100.8–114.1)	0.27
Blood urea nitrogen (7–27 mg/dL)	7–27	24.5 (8.0–40.0)	24.0 (12.0–41.0)	0.82
Albumin (2.6–2–3.91 g/dL)	2.62.3.91	3.13 (2.67–3.74)	3.36 (2.59–3.79	0.30
Calcium (8.7–10.4 mg/dL)	8.7–10.4	10.2 (8.8–11.2)	10.3 (9.4–10.9)	1.0
Creatinine (0.6–1.5 mg/dL)	0.6–1.5	0.99 (0.58–1.46)	0.85 (0.64–1.32)	0.37
Phosphorous (2.2–4.8 mg/dL)	2.2–4.8	4.4 (2.1–6.4)	4.2 (3.2–4.9)	0.17
ACE activity (ng/mL)		178.7 (95.9–262.0)	172.2 (92.4–261.6)	0.46
ACE2 activity (ng/mL)		77.5 (20.7–238.6)	68.7 (24.7–221.0)	0.99
NEP (ng/mL)		7.13 (1.12–58.87)	8.09 (1.20–22.03)	0.24
Angiotensin I (pmol/L)		563.0 (84.7–2572.0)	298.4 (87.6–2024)	0.15
Angiotensin II (pmol/L)		238.7 (12.6–1281.0)	145.0 (20.1–1222.0)	0.24
Angiotensin III (pmol/L)		26.2 (1.3–83.5)	11.0 (1.3–136.3)	0.19
Angiotensin IV (pmol/L)		38.5 (1.0–154.6)	15.5 (5.8–193.7)	0.19
Angiotensin 1–5 (pmol/L)		243.3 (9.9–949.9)	208.8 (66.5–1213.0)	0.24
Angiotensin 1–7 (pmol/L)		247.7 (46.1–1369.0)	153.8 (42.8–941.1)	0.15
Aldosterone (pmol/L)		295.6 (7.0–1299.0)	70.5 (5.0–1069.0)	0.06
AA2		1.00 (0.03–7.05)	0.65 (0.01–3.63)	**0.009**
PRA-S (pmol)		850.1 (107.7–3854.0)	447.9 (127.4–3246.0)	0.17
ACE-S		0.50 (0.08–1.01)	0.55 (0.13–0.74)	0.94
ALT-S		0.56 (0.32–0.90)	0.65 (0.29–0.95)	0.10
Angiotensin 1–7/Angiotensin 1		0.96 (0.38–6.23)	1.01 (0.51–4.30)	0.82

**Figure 1:**
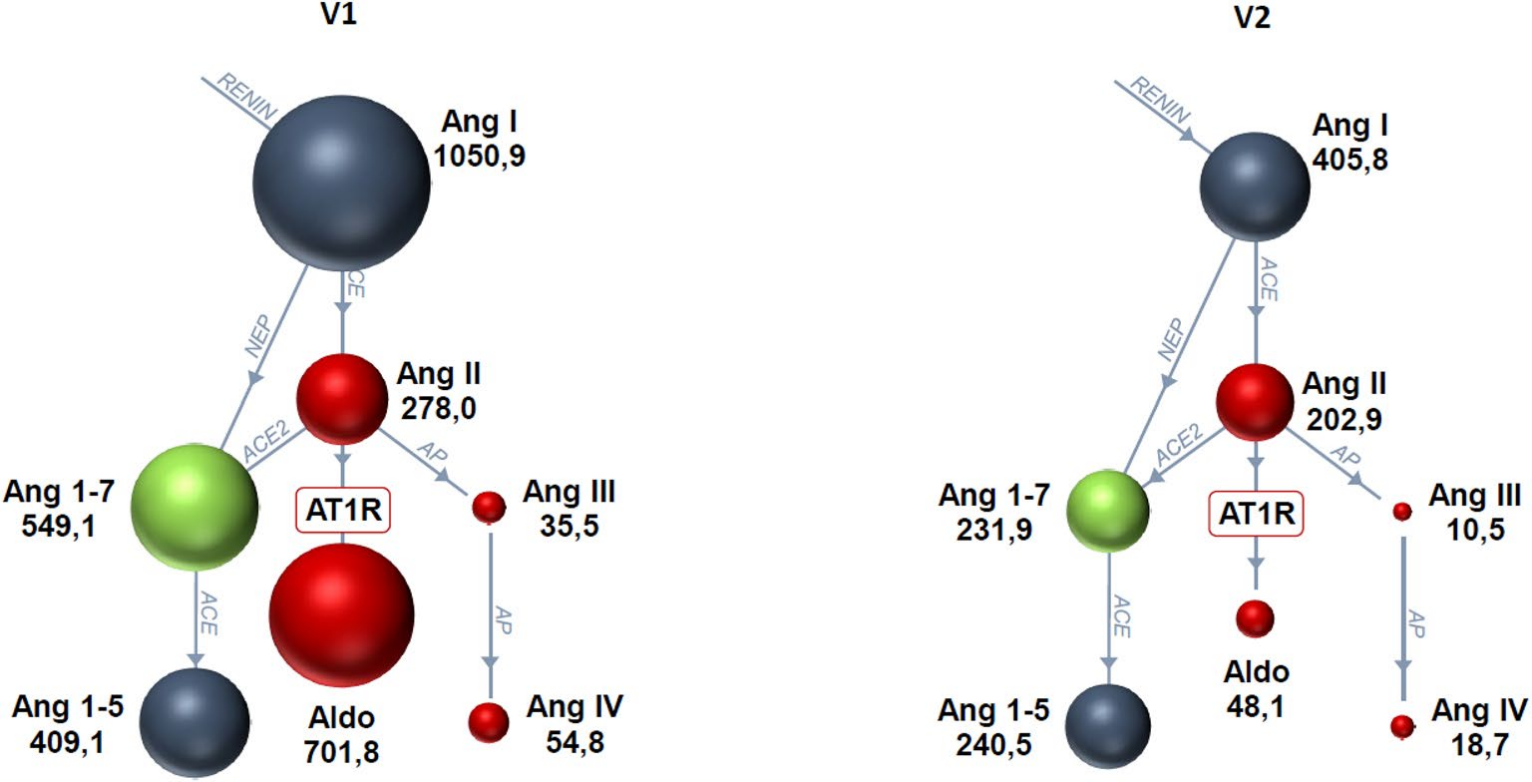
Renin–angiotensin–aldosterone system (RAAS) Fingerprints™ at visit 1 (V1) and visit 2 (V2). The size of the sphere indicates the median metabolite value (pmol/L) for the group of dogs for each metabolite. Red spheres indicate classical RAAS metabolites that mediate vasoconstriction and sodium retention, green spheres indicate alternative RAAS metabolites that mediate vasodilation and natriuresis, and blue spheres indicate inert metabolites. Ang 1; angiotensin I, Ang II; angiotensin II, Ang III; angiotensin III, Ang IV; angiotensin IV, AT1R; angiotensin receptor type 1, Ang 1–7; angiotensin 1–7, Ang 1–5; angiotensin 1–5, aldo; aldosterone.

**Figure 2:**
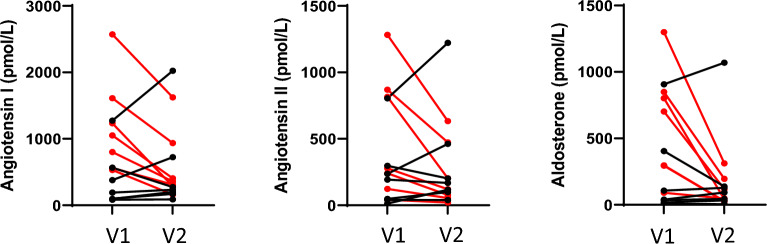
Angiotensin I, angiotensin II, and aldosterone values are shown for each dog at each time point with connecting lines to indicate the same dog. Dots and lines colored red indicate dogs that were hospitalized for treatment of congestive heart failure within 1 week of enrollment. Dots and lines colored black indicate dogs that were hospitalized for treatment of congestive heart failure more than 1 week before enrollment. V1; visit 1 (before taurine supplementation), V2; visit 2 (2 weeks after taurine supplementation).

The only RAAS variable that showed a statistically significant change was AA2, which decreased between visits (*P* = 0.009). Most other RAAS metabolites decreased between timepoints but the differences did not reach statistical significance (Table 1 and Fig. 1). Seven dogs were hospitalized for the treatment of CHF < 1 week before enrollment and 7 dogs were hospitalized for the treatment of CHF > 1 week before enrollment. Figure 2 shows the individual dog data for angiotensin I, angiotensin II and aldosterone color-coded according to whether dogs were recently hospitalized for the treatment of CHF. All but 1–2 of the highest individual values for angiotensin I, angiotensin II, and aldosterone at V1 were from dogs that were hospitalized within 1 week. Five dogs were classified as RAAS responders and 9 as non-responders. Responders were not more likely than non-responders to be ACE variant positive (*P* = 0.58) but were more likely to have been discharged from the hospital within a week of enrollment (*P* = 0.02).

The activity of ACE was significantly lower in ACE variant positive dogs compared to ACE wildtype dogs (*P* = 0.005) but the ACE-S was not different between genotype groups (*P* = 0.35). The activity of ACE2 and NEP were not different between genotype groups (*P* > 0.35).

## Discussion

The results of this study were not supportive of a RAAS suppressive effect of oral taurine supplementation for this group of dogs with naturally occurring CHF secondary to mitral valve degeneration. Nearly all the RAAS metabolites decreased, but the only variable that was statistically significantly different between visits was AA2, which reflects the relationship between aldosterone and angiotensin II and is typically viewed as an indicator of adrenal responsiveness to angiotensin II. The AA2 ratio was significantly lower at V2 because a larger decrease in aldosterone occurred compared to angiotensin II. The decrease in aldosterone between visits was notable, but did not reach statistical significance. The clinical significance of this is unknown, but it is possible that non-angiotensin II sources of aldosterone stimulation (e.g. direct beta stimulation) were suppressed by taurine^26^.

Despite the lack of significant differences in RAAS variables after 2 weeks of oral taurine supplementation, a subset of dogs showed decreases in classical RAAS metabolites that were greater than expected for temporal variability, suggesting response heterogeneity within this group of dogs. The decreases in classical metabolite in this subset of dogs were not consistent with an ACE-inhibitory effect, but were global and characterized by decreases in angiotensin I, angiotensin II, and aldosterone which could be a result of adrenergic antagonism as shown in other studies^19,27^. An ACE-inhibitory effect would have been characterized by decreases in angiotensin II and aldosterone and an increase in angiotensin I. Supportive of this was the lack of observable effect of the ACE variant on whether dogs showed decreases in classical RAAS metabolites.

Dogs showing RAAS suppression were more likely to have been recently hospitalized for the treatment of CHF, and these dogs had some of the highest baseline values for classical RAAS metabolites. Dogs with naturally occurring CHF were selected for this study to provide a background of intense RAAS activation due to the presence of advanced heart disease, administration of diuretics, and absence of RAAS-suppressive medications such as ACE-inhibitors, angiotensin-receptor blockers, or mineralocorticoid antagonists. This finding suggests that recent hospitalization might be a cause of even greater RAAS activation for dogs with advanced heart disease and CHF. Contributing factors could include stress, the presence of active CHF, and administration of intravenous diuretic therapy with accompanying volume and electrolyte depletion. The higher body weight at V1 compared to V2 is unexplained, but does not support volume depletion at the initial visit relative to the recheck. One study found that some people with CHF had greater degrees of RAAS activation than other people and this was not related to whether they received low or high doses of diuretic in the hospital^28^. The degree of RAAS activation in our study at V1 was similar to that produced in healthy dogs with the administration of loop diuretics by different routes^29,30^. Additionally, the change in clinical variables (e.g. body weight, heart rate, blood pressure) and biochemical variables reflecting volume status (e.g. albumin, blood urea nitrogen, creatinine) between visits, did not support the presence of significant volume depletion at V1 that was corrected at V2. The increase in serum sodium could be a result of the transition from intravenous to oral diuretic therapy or might be a result of the observed RAAS suppression as has been noted in people^28^. Regardless of the cause, higher RAAS activation in some dogs at V1 might have contributed to the notable decline in classical RAAS metabolites after taurine supplementation. This provides insight into the mechanism of taurine benefit through global RAAS suppression and similar to studies in hypertensive rats, suggests that benefit might be best realized in patients with the most baseline RAAS activation^27^.

Consistent with previous studies of the known ACE variant in dogs, we found that ACE activity was significantly lower in ACE gene variant positive dogs compared to ACE gene wildtype dogs^31^. The lack of difference in ACE-S (a proposed surrogate for ACE activity) between ACE variant and wildtype dogs has also been previously reported^32,33^. Because ACE-S is simply the ratio of angiotensin II to angiotensin I, it appears not to reflect true ACE activity in dogs with the ACE variant because other non-ACE enzymes (e.g. chymase) can form angiotensin II when ACE activity is decreased. Our results did not support an ACE-inhibitory effect even in the subset of dogs that appeared to respond, and so it is not surprising that the ACE variant did not affect the results. Other RAAS polymorphisms could be present in dogs with heart disease.

The activity of ACE2 has not been previous reported in dogs with CHF. The ACE2 activity for this group of CHF dogs was notably higher than reported for both healthy people and people with CHF^34^ but was not markedly different from healthy dogs^33^. This enzyme is a marker for disease severity in people with heart disease. More work is needed to understand ACE2 in naturally occurring heart disease in dogs but our data supports notable species differences. Ours is also the first study to report NEP activity in dogs although we did not find a difference between visits for this enzyme.

This study has several limitations. Although a priori power analysis indicated a minimum of 12 dogs was needed to show group differences in angiotensin II, our study can still be considered small. The power to detect group differences could have been impacted by unknown factors introducing important variability, including a more advanced stage of heart disease for the included dogs compared to dogs used for sample size calculation. Recent hospitalization for CHF treatment appeared to influence the decrease in classical RAAS activation for a subset of dogs in our study, even though the overall degree of RAAS activation for the group at V1 was similar to previous reports of dogs receiving oral diuretics^29,30^. The use of dogs as their own control, therefore is a weakness of this study (even though week-to-week variability for these metabolites has been reported^35^), whereas the use of a group receiving placebo would have controlled for confounders such as recent hospitalization. The decreases in most RAAS metabolites did not reach statistical significance between timepoints; while this might indicate a true finding, it could be due to low power resulting from high measurement variability. Evaluation of the effect of taurine in dogs that are naturally RAAS activated and not receiving RAAS inhibiting medications was a strength of this study. Additionally, we documented an increase in whole blood taurine concentration that was well above the reference range after supplementation, although indirect benefits of taurine supplementation might take longer than 2 weeks to be realized (e.g. indirect decreases in RAAS through improved myocardial function).

## Conclusions

In conclusion, although we did not find a conclusive RAAS suppressive effect of taurine supplementation in this group of dogs with naturally occurring CHF treated with diuretic therapy and withheld from RAAS suppressive medications, AA2 decreased significantly after supplementation as a result of a greater decrease in aldosterone compared to angiotensin II. Additionally, there was response heterogeneity within this group of dogs, with some showing marked decreases in RAAS metabolite values after supplementation. Other mechanisms through which taurine might benefit dogs with heart disease who are not taurine deficient should be explored in future studies.

## Methods

This prospective study was approved by the Institutional Animal Care and Use Committee at the University of Florida College of Veterinary Medicine (#MS1-201910865 and 202200000396) and the authors complied with the ARRIVE guidelines. All methods were performed in accordance with the relevant guidelines and regulations. All dog owners provided informed consent.

### Inclusion criteria

Dogs being treated for naturally occurring CHF secondary to degenerative mitral valve disease (American College of Veterinary Internal Medicine Stage C)^3^ were included if they were stable on oral furosemide and pimobendan and did not have active radiographic or clinical signs of uncontrolled CHF. Dogs with the underlying condition of degenerative mitral valve disease were targeted for enrollment instead dogs with dilated cardiomyopathy due to the higher frequency of degenerative mitral valve disease in the canine population. Both conditions lead to the same end result of CHF with common pathophysiology. Dogs were eligible for enrollment after being treated in the hospital for left-sided CHF once they were transitioned to oral medications. A body weight greater than 4 kg was required to facilitate blood sampling.

### Exclusion criteria

Dogs that received RAAS-suppressive therapies (e.g. ACE-inhibitors, angiotensin receptor blockers, spironolactone) or taurine supplementation within the previous 2 weeks were excluded. Dogs with right-sided CHF were excluded.

### Procedures

#### Timeline

Buccal swab and blood (8 mLs) collected by peripheral venipuncture were obtained at V1 for biochemical analysis, RAAS analysis, taurine concentrations and ACE genotyping. Taurine supplementation (Now® or PetAg® brands) was started after V1 (target daily dosage 60 mg/kg/day divided into 2 doses 12 h apart, dose was rounded up to fit tablet or capsule sizes) in addition to current cardiac medications. Blood samples were collected again 2 weeks later (V2) for biochemical analysis, RAAS analysis, and taurine concentrations.

#### Collection of clinical data

Breed, age, sex, body weight, systolic blood pressure (by Doppler), heart rate, medication dosages, and time (< or > 1 week) from last hospitalization for CHF were recorded for each dog.

Serum biochemical analysis: Five mLs of blood were placed into additive-free tubes and centrifuged at 3000 rpm for 10 min. Serum was removed and placed into 2 aliquots. One aliquot was submitted to the University of Florida Clinical Pathology Laboratory for biochemical analysis (blood urea nitrogen, creatinine, albumin, sodium chloride, potassium, calcium, phosphorous, anion gap). The 2nd aliquot was stored at −80 °C until batch analysis for RAAS analysis.

#### Blood taurine concentrations

Three mLs of blood were placed into 2 lithium heparin tubes, one of which was centrifuged at 3000 rpm for 10 min for plasma removal. The plasma sample and the whole blood sample were shipped on dry ice to the University of California, Davis, Amino Acid Laboratory (Davis, CA) for measurement of whole blood and plasma taurine concentrations.

#### Genotyping

The buccal swab was used to genotype dogs for the known ACE variant which can affect baseline ACE activity and aldosterone breakthrough^32^. Samples were sent for analysis to the North Carolina State University Veterinary Genetics Laboratory (https://cvm.ncsu.edu/nc-state-vet-hospital/small-animal/genetics/submit-dna-testing/). Nucleotide sequences were visually evaluated for sequence quality and aligned using sequence analysis software to determine the presence or absence of the ACE gene polymorphism.

#### Equilibrium analysis of the RAAS

This study uses the methods described in previous studies and this methods description partly reproduces their wording^32–34^. The equilibrium concentrations of 6 different angiotensin peptide metabolites (angiotensin I, angiotensin II, angiotensin III, angiotensin IV, angiotensin 1–5, angiotensin 1–7) and aldosterone were quantified by liquid chromatography-mass spectrometry/mass-spectroscopy (LC–MS/MS), performed by a service provider laboratory (Attoquant Diagnostics, Vienna, Austria), using previously validated and described methods^14^. Analyte concentrations were reported in pmol/L and the lower limit of quantification was reported for each metabolite. The activities of ACE and ACE2 were measured with a kinetics approach using spiked substrate^33,36^. Activity of ACE was determined by measuring the formation of angiotensin II after addition of angiotensin I in the presence and absence of an ACE inhibitor and a chymase inhibitor to determine the ACE-inhibitor sensitive fraction of angiotensin II generation. Activity of ACE2 was determined by measuring the formation of angiotensin 1–7 after the addition of angiotensin II in the presence and absence of an ACE2 inhibitor using recombinant human ACE2 as a reference standard. Activity of neutral endopeptidase (NEP) was determined by measuring the formation of angiotensin 1–7 after addition of angiotensin I in the presence and absence of a NEP inhibitor. The ratio of angiotensin II to angiotensin I was calculated as a marker for ACE activity (ACE-S)^32^. Angiotensin I and angiotensin II were summed as a marker for plasma renin activity (PRA-S)^37^. The ratio of aldosterone to angiotensin II (AA2) was calculated as a unitless indicator of adrenal responsiveness to angiotensin II stimulation of aldosterone release^32^. The sum of angiotensins 1–7 and 1–5 divided by the sum of angiotensins I, II, 1–7, and 1–5, was calculated as a unitless marker of renin-independent alternative RAAS activation (ALT-S)^33^. The ratio of angiotensin 1–7/angiotensin I was calculated to account for overall RAAS activity to aid interpretation of the balance of angiotensin 1–7 formation and breakdown^33^.

#### Statistical analysis

A priori power analysis showed a minimum of 12 dogs would be needed to show a significant decrease in angiotensin II concentrations between V1 and V2 with a power of 80% and significance level of 5%, using the decrease in angiotensin II after enalapril administration in a previous study of dogs with preclinical mitral valve disease for this calculation (mean 43 pM ± standard deviation 47 pM)^32^. Clinical data, biochemical data, RAAS data, and taurine data were assessed for normality using Shapiro–Wilk test, and median (range) were reported for each visit (V1, V2). Variables were compared between V1 and V2 using Wilcoxon matched-pairs signed rank test. Individual dogs were considered RAAS responsive if they showed a decrease in at least 3 classical RAAS components (angiotensin I, angiotensin II, angiotensin III, angiotensin IV, or aldosterone) of > 55% to account for temporal variability at V2^35^. Fisher’s exact test was used to assess if ACE genotype or time from CHF episode (< 1 week, > 1 week) was associated with being RAAS responsive. The activity of ACE, ACE2, and NEP were compared between ACE genotypes using Mann–Whitney test. Metabolite concentrations below the lower limit of quantification were entered as half the lower limit of quantification for statistical comparisons. Commercially available statistical software was used for analysis (GraphPad Prism 9). *P* < 0.05 was considered statistically significant.

### Institutional animal care and use committee (IACUC) or other approval declaration

This study was approved by the Institutional Care and Use Committee at the University of Florida, College of Veterinary Medicine (#MS1-201910865 and 202200000396).

### Human ethics approval declaration

Authors declare human ethics approval was not needed for this study.

## Supplementary Information

Supplementary Table S1.

Supplementary Legends.

## Data Availability

All data generated or analysed during this study are included in this published article (and its Supplementary Information file).

## Abbreviations

AA2: Aldosterone to angiotensin II ratio
ACE: Angiotensin-converting enzyme
ACE2: Angiotensin-converting enzyme 2
ACE-S: Angiotensin-converting enzyme surrogate
ALT-S: Alternative RAAS activation marker
CHF: Congestive heart failure
NEP: Neutral endopeptidase
PRA-S: Plasma renin activity marker
RAAS: Renin–angiotensin–aldosterone system

## References

[1] Borgarelli M, Buchanan JW (2012). Historical review, epidemiology and natural history of degenerative mitral valve disease. J. Vet. Cardiol..

[2] Wess G, Domenech O, Dukes-McEwan J, Häggström J, Gordon S (2017). European society of veterinary cardiology screening guidelines for dilated cardiomyopathy in Doberman Pinschers. J. Vet. Cardiol..

[3] Keene BW, Atkins CE, Bonagura JD, Fox PR, Fuentes VL, Uechi M (2019). ACVIM consensus guidelines for the diagnosis and treatment of myxomatous mitral valve disease in dogs. J. Vet. Intern. Med..

[4] Schaffer S, Kim HW (2018). Effects and mechanisms of taurine as a therapeutic agent. Biomol. Ther. (Seoul).

[5] Lourenco R, Camilo ME (2002). Taurine: A conditionally essential amino acid in humans? An overview in health and disease. Nutr. Hosp..

[6] Belanger M, Ouetllet M, Queney G, Moreau M (2005). Taurine-deficient dilated cardiomyopathy in a family of golden retrievers. J. Am. Anim. Hosp. Assoc..

[7] Kittleson MD, Keene B, Pion PD, Loyer CG (1997). Results of the multicenter spaniel trial (MUST): Taurine- and carnitine-responsive dilated cardiomyopathy in American cocker spaniels with decreased plasma taurine concentration. J. Vet. Intern. Med..

[8] Pion PD, Kittleson MD, Skiles ML, Rogers QR, Morris JG (1992). Dilated cardiomyopathy associated with taurine deficiency in the domestic cat: Relationship to diet and myocardial taurine content. Taurine..

[9] Backus R, Ko K, Fascetti A, Kittleson M, MacDonald K, Maggs D (2006). Low plasma taurine concentration in Newfoundland dogs is associated with low plasma methionine and cysteine concentrations and low taurine synthesis. J. Nutr..

[10] Ontiveros ES, Whelchel BD, Yu J, Kaplan JL, Sharpe AN, Fousse SL (2020). Development of plasma and whole blood taurine reference ranges and identification of dietary features associated with taurine deficiency and dilated cardiomyopathy in golden retrievers: A prospective, observational study. PLoS ONE.

[11] Fascetti AJ, Reed JR, Rogers QR, Backus RC (2003). Taurine deficiency in dogs with dilated cardiomyopathy: 12 cases (1997–2001). J. Am. Vet. Med. Assoc..

[12] Haimovitz D, Vereb M, Freeman L, Goldberg R, Lessard D, Rush J (2022). Effect of diet change in healthy dogs with subclinical cardiac biomarker or echocardiographic abnormalities. J. Vet. Intern. Med..

[13] Adin D, Freeman L, Kellihan H, Stepien R, Aherne M, Rush JE (2021). Effect of type of diet on blood and plasma taurine concentrations, cardiac biomarkers, and echocardiograms in 4 dog breeds. J. Vet. Intern. Med..

[14] Smith CE, Parnell LD, Lai CQ, Rush JE, Freeman LM (2021). Investigation of diets associated with dilated cardiomyopathy in dogs using foodomics analysis. Sci. Rep..

[15] Freid KJ, Freeman LM, Rush JE, Cunningham SM, Davis MS, Karlin ET (2021). Retrospective study of dilated cardiomyopathy in dogs. J. Vet. Intern. Med..

[16] Kaplan JL, Stern J, Fascetti AJ, Larsen JA, Skolnik H, Peddle GD (2018). Taurine deficiency and canine dilated cardiomyopathy in golden retrievers fed commercial diets. PLoS ONE.

[17] Walker AL, DeFrancesco TC, Bonagura JD, Keene BW, Meurs KM, Tou SP (2022). Association of diet with clinical outcomes in dogs with dilated cardiomyopathy and congestive heart failure. J. Vet. Cardiol..

[18] Adin D, DeFrancesco TC, Keene B, Tou S, Meurs K, Atkins C (2019). Echocardiographic phenotype of canine dilated cardiomyopathy differs based on diet type. J. Vet. Cardiol..

[19] Ito T, Schaffer S, Azuma J (2014). The effect of taurine on chronic heart failure: Actions of taurine against catecholamine and angiotensin II. Amino Acids.

[20] Yan-rong SHI, Ding-fang BU, Yong-fen QI, Lin GAO, Hong-feng J (2002). Dysfunction of myocardial taurine transport. Acta Pharmacol. Sin..

[21] Xu YJ, Arneja AS, Tappia PS (2008). Experimental cardiology: Review: The potential health benefits of taurine in cardiovascular disease. Exp. Clin. Cardiol..

[22] Fujita T, Ando K, Noda H, Ito Y, Sato Y (1987). Effects of increased adrenomedullary activity and taurine in young patients with borderline hypertension. Circulation.

[23] Azuma J, Sawamura A, Awata N, Ohta H, Hamaguchi T, Harada H (1985). Therapeutic effect of taurine in congestive heart failure: A double-blind crossover trial. Clin. Cardiol..

[24] Larouche-Lebel É, Loughran KA, Oyama MA, Solter PF, Laughlin DS, Sánchez MD (2019). Plasma and tissue angiotensin-converting enzyme 2 activity and plasma equilibrium concentrations of angiotensin peptides in dogs with heart disease. J. Vet. Intern. Med..

[25] Adin D, Kurtz K, Atkins C, Papich MG, Vaden S (2020). Role of electrolyte concentrations and renin-angiotensin-aldosterone activation in the staging of canine heart disease. J. Vet. Intern. Med..

[26] de Lean A, Karoly R, McNicoll N, LuceDesrosiers M (1984). Direct beta-adrenergic stimulation of aldosterone secretion in cultured bovine adrenal subcapsular cells. Endocrinology.

[27] Yamamoto J, Akabane S, Yoshimi H, Nakai M, Ikeda MA (1985). Effects of taurine on stress-evoked hemodynamic and plasma catecholamine changes in spontaneously hypertensive rats. Hypertens. [Internet]..

[28] Mentz RJ, Stevens SR, DeVore AD, Lala A, Vader JM, AbouEzzeddine OF (2015). Decongestion strategies and renin-angiotensin-aldosterone system activation in acute heart failure. JACC Heart Fail..

[29] Potter BM, Ames MK, Hess A, Poglitsch M (2019). Comparison between the effects of torsemide and furosemide on the renin-angiotensin-aldosterone system of normal dogs. J. Vet. Cardiol..

[30] Harada K, Ukai Y, Kanakubo K, Yamano S, Lee J, Kurosawa TA (2015). Comparison of the diuretic effect of furosemide by different methods of administration in healthy dogs. J. Vet. Emerg. Crit. Care.

[31] Meurs KM, Stern JA, Atkins CE, Adin D, Aona B, Condit J (2017). Angiotensin-converting enzyme activity and inhibition in dogs with cardiac disease and an angiotensin-converting enzyme polymorphism. J. Renin Angiotensin Aldosterone Syst..

[32] Adin D, Atkins C, Domenig O, DeFrancesco T, Keene B, Tou S (2020). Renin-angiotensin aldosterone profile before and after angiotensin-converting enzyme-inhibitor administration in dogs with angiotensin-converting enzyme gene polymorphism. J. Vet. Intern. Med..

[33] Adin DB, Hernandez JA (2022). Influence of sex on renin-angiotensin-aldosterone system metabolites and enzymes in Doberman Pinschers. J. Vet. Intern. Med..

[34] Basu R, Poglitsch M, Yogasundaram H, Thomas J, Rowe BH, Oudit GY (2017). Roles of angiotensin peptides and recombinant human ACE2 in heart failure. J. Am. Coll. Cardiol..

[35] Hammond HH, Ames MK, Domenig O, Scansen BA, Tsang Yang N, Wilson MD (2023). The classical and alternative circulating renin-angiotensin system in normal dogs and dogs with stage B1 and B2 myxomatous mitral valve disease. J. Vet. Intern. Med..

[36] Kintscher, U., Slagman, A., Domenig, O., Röhle, R., Konietschke, F., Poglitsch, M. *et al*. Plasma angiotensin peptide profiling and ACE (angiotensin-converting enzyme)-2 activity in COVID-19 patients treated with pharmacological blockers of the renin-angiotensin system. *Hypertension*. E34–6 (2020). 10.1161/HYPERTENSIONAHA.120.15841 PMC7480797 32851897

[37] Pavo N, Goliasch G, Wurm R, Novak J, Strunk G, Gyöngyösi M (2018). Low-and high-renin heart failure phenotypes with clinical implications. Clin. Chem..

