# Supplementary material for: The effect of taurine supplementation on the renin–angiotensin–aldosterone system of dogs with congestive heart failure
Source: Sci Rep. 2023 Jul 3;13:10700. doi: 10.1038/s41598-023-37978-1 (PMC10318075; doi:10.1038/s41598-023-37978-1)
Supplement: Supplementary file 2 — Supplementary Legends. [file 41598_2023_37978_MOESM2_ESM.docx]

**Supplemental Table 1.** Raw data for the study is provided in this table
